# Case Report: persistent diaphragmatic and cardiac involvement despite creatine kinase normalization in anti-mitochondrial M2 antibody-positive inflammatory myopathy with primary biliary cholangitis

**DOI:** 10.3389/fimmu.2026.1961744

**Published:** 2026-10-09

**Authors:** Zonghui Hou, Zhijin Xu, Lianyi Wang

**Affiliations:** 1 Department of Cardiology, The First Hospital of Tsinghua University, School of Clinical Medicine, Tsinghua Medicine, Tsinghua University, Beijing, China; 2 Department of Oncology (Radiotherapy and Chemotherapy), The First Affiliated Hospital of Wenzhou Medical University, The First School of Clinical Medicine, School of Information and Engineering, Wenzhou Medical University, Wenzhou, Zhejiang, China

**Keywords:** AMA-M2, anti-HMGCR, cardiac involvement, creatine kinase, diaphragmatic dysfunction, inflammatory myopathy, primary biliary cholangitis, respiratory muscle weakness

## Abstract

Anti-mitochondrial M2 antibodies (AMA-M2) are a serologic hallmark of primary biliary cholangitis (PBC), but in inflammatory myopathy they have also been associated with chronic muscle disease, cardiac involvement, and respiratory muscle weakness. We report a 44-year-old woman who developed progressive proximal lower-limb weakness followed by palpitations, exertional dyspnea, and severe hypercapnic respiratory failure. Serum creatine kinase (CK) was only moderately elevated (peak, 789 U/L). AMA-M2 remained strongly positive with anti-gp210 and anti-sp100 antibodies. Low-level anti-HMGCR IgG was detected on two occasions (0.35 and 0.27 U/mL; positivity cutoff >0.15 U/mL), whereas anti-SRP was negative. Electromyography showed myopathic changes with abundant myotonic discharges, and thigh magnetic resonance imaging demonstrated inflammatory signal abnormalities with muscle atrophy and fatty infiltration. An outside muscle biopsy was recorded as favoring dermatomyositis, but the original report and slides could not be retrieved and repeat biopsy was declined. Without independently reviewable biopsy data, the 2017 EULAR/ACR score was 7.0 (estimated IIM probability, 62.5%), meeting the threshold for probable IIM. Arterial carbon dioxide tension reached 94.6 mmHg. Chest imaging showed no extensive parenchymal disease sufficient to explain the degree of hypoventilation, whereas serial diaphragm ultrasonography demonstrated marked dysfunction. Cardiac evaluation revealed conduction abnormalities, atrial and ventricular arrhythmias, elevated high-sensitivity cardiac troponin I (hs-cTnI) and B-type natriuretic peptide (BNP), regional wall-motion abnormality, and reduced global longitudinal strain despite preserved left ventricular ejection fraction. After glucocorticoids, intravenous immunoglobulin, noninvasive ventilation, and individualized immunotherapy, CK normalized earlier than the cardiac and respiratory abnormalities. At approximately 4 months, CK and hs-cTnI were normal, but FVC remained 51% predicted, TLC 57.5% predicted, bilateral diaphragm thickening fractions 10%-11%, and mild hypercapnia persisted. At approximately 7 months, cardiac abnormalities remained evident. These asynchronous trajectories indicate that CK normalization may underestimate ongoing organ burden and support organ-specific surveillance during follow-up.

## 1 Introduction

AMA-M2 is best known as a serologic marker of PBC, but its significance in autoimmune myositis is increasingly recognized. Early descriptions of AMA-positive inflammatory myopathy emphasized a chronic course, muscle atrophy, cardiac abnormalities, and reduced vital capacity, with heterogeneous muscle pathology (1–4). A large clinicopathologic study published in 2025 further proposed that AMA-M2-positive myositis may represent a distinct autoimmune myositis phenotype characterized by chronic myopathy, partial overlap with immune-mediated necrotizing myopathy-like pathology, and a relatively high frequency of cardiac and respiratory muscle involvement (5).

These organ manifestations are clinically important. In a 17-patient series, respiratory failure and cardiac complications were prominent and vital capacity was proposed as a longitudinal marker (6). A subsequent nationwide Japanese survey found cardiac complications in 53%, restrictive ventilatory impairment in 33%, and a need for respiratory support in 22% of patients (7). Severe respiratory failure may also occur early, before limb weakness or CK elevation becomes striking (8, 9). In a recent cohort of 987 patients with idiopathic inflammatory myopathy (IIM), cardiac involvement occurred in 60% of AMA-M2-positive patients and arrhythmia in 56%; AMA-M2 positivity was independently associated with cardiac involvement (10). A separate analysis of 123 adults with AMA-positive myositis likewise showed a substantial cardiac burden (11).

Most reports have focused on whether cardiac or respiratory muscle involvement occurs. Less attention has been paid to whether these manifestations recover in parallel with conventional skeletal muscle biomarkers after treatment. In the present case, serial arterial blood gases, diaphragm ultrasonography, pulmonary function testing, ambulatory electrocardiography, cardiac biomarkers, and myocardial strain imaging documented a clear temporal dissociation: CK normalized early, while clinically relevant diaphragmatic and cardiac abnormalities followed different and slower recovery trajectories.

## 2 Case description

### 2.1 Clinical history and initial assessment

A 44-year-old woman with no smoking or alcohol history and no known neuromuscular disease or cardiomyopathy developed bilateral proximal lower-limb weakness approximately 7 months before the index admission. Difficulty climbing stairs was the main limitation, although she remained independently ambulatory. She reported no myalgia, arthralgia, dysphagia, or typical dermatomyositis rash. The principal clinical events and treatments are summarized in **Table 1**.

**Table 1:** Clinical timeline.

Relative time (T0 = index admission)	Key clinical findings	Management/outcome
T - ~7 months	Progressive bilateral proximal lower-limb weakness; CK 785 U/L; hs-cTnI 0.487 ng/mL (outside reference range 0-0.0387); atrial/ventricular ectopy, short tachyarrhythmias and atrioventricular conduction abnormalities; abnormal liver tests; AMA-M2, anti-sp100 and anti-gp210 positive.	First systematic evaluation at an outside hospital.
T - ~2 months	Persistent weakness with palpitations and exertional dyspnea; CK 789 U/L; hs-cTnT 144 pg/mL; moderate-to-severe restrictive ventilatory defect; first-degree and second-degree Mobitz I atrioventricular block; regional wall-motion abnormality.	Short course of methylprednisolone at an outside hospital; limited improvement.
T - ~7 weeks to 1 month	Severe hypercapnic respiratory failure (PaCO2 up to 94.6 mmHg); thigh MRI with inflammatory abnormalities and atrophy/fatty infiltration; myopathic electromyography with abundant myotonic discharges; predominantly right-sided diaphragmatic dysfunction; reduced GLS; AMA-M2 >200 RU/mL with PBC-related antibodies.	Methylprednisolone, IVIG, brief mycophenolate mofetil and NIV; PaCO2 and CK gradually decreased.
T0 to T + ~3 weeks	MMT8 76/80; neck flexion MRC 4/5; gluteus maximus 4/5; proximal lower-limb weakness greater than distal; persistent diaphragmatic dysfunction; preserved LVEF but regional wall-motion abnormality and reduced GLS; frequent PAC/PVC and short atrial tachycardia; hs-cTnI/BNP remained abnormal after CK normalized.	Tacrolimus stopped because of worsening cholestatic liver tests; rituximab 600 mg IV at approximately T + 1 and T + 3 weeks; glucocorticoids tapered.
T + ~4 months	CK 161 U/L and hs-cTnI 0.0048 ng/mL; BNP 194 pg/mL; residual mild hypercapnia; FVC 51% predicted, TLC 57.5% predicted; bilateral diaphragm TF 10%-11%; CD19-positive B cells 0.1%.	Home NIV still used; glucocorticoid taper and PBC treatment continued.
T + ~7 months	BNP 187.52 pg/mL; echocardiography showed persistent basal inferoposterior wall hypokinesis and grade II diastolic dysfunction; CD19-positive B cells 0%.	Further rituximab 600 mg IV; prednisone 15 mg/day continued. No repeat PFT or arterial blood gas was available at this admission.

AMA-M2, anti-mitochondrial M2 antibody; BNP, B-type natriuretic peptide; CK, creatine kinase; GLS, global longitudinal strain; hs-cTnI, high-sensitivity cardiac troponin I; hs-cTnT, high-sensitivity cardiac troponin T; IVIG, intravenous immunoglobulin; LVEF, left ventricular ejection fraction; MMT8, manual muscle testing 8; NIV, noninvasive ventilation; PAC, premature atrial contraction; PBC, primary biliary cholangitis; PFT, pulmonary function testing; PVC, premature ventricular contraction; TF, diaphragm thickening fraction.

Approximately 7 months before admission, serum CK was 785 U/L at an outside hospital. The outside record documented hs-cTnI of 0.487 ng/mL (reference range, 0-0.0387 ng/mL). Ambulatory electrocardiography showed frequent atrial and ventricular ectopy, short tachyarrhythmic runs, and atrioventricular conduction abnormalities. Liver tests were also abnormal: alanine aminotransferase 177.8 U/L, aspartate aminotransferase 120 U/L, gamma-glutamyl transferase 305 U/L, and alkaline phosphatase 361 U/L. Antinuclear antibodies, AMA-M2, anti-sp100, and anti-gp210 antibodies were positive. Transthoracic echocardiography showed preserved global systolic function with only mild valvular regurgitation.

Approximately 2 months before admission, weakness persisted and palpitations and exertional dyspnea became more prominent. Repeat testing at another hospital showed CK 789 U/L and hs-cTnT 144 pg/mL. Pulmonary function testing showed a moderate-to-severe restrictive ventilatory defect. Ambulatory electrocardiography documented first-degree and second-degree Mobitz I atrioventricular block, and echocardiography showed a regional left ventricular wall-motion abnormality. A short course of methylprednisolone 24 mg/day for approximately 6 days produced limited symptomatic improvement and was not continued after discharge.

### 2.2 Diagnostic assessment: AMA-M2-positive inflammatory myopathy and PBC

At the index admission, weakness predominantly involved the proximal lower limbs, which were clearly weaker than the distal muscles. Neck flexion was Medical Research Council grade 4/5 and weaker than neck extension; gluteus maximus strength was grade 4/5. MMT8 was 76/80. Electromyography showed myopathic changes in the tibialis anterior, biceps brachii, quadriceps, and iliopsoas with abundant myotonic discharges. Thigh MRI showed bilateral inflammatory signal abnormalities with muscle atrophy and fatty infiltration (**Figures 1A, B**).

**Figure 1:**
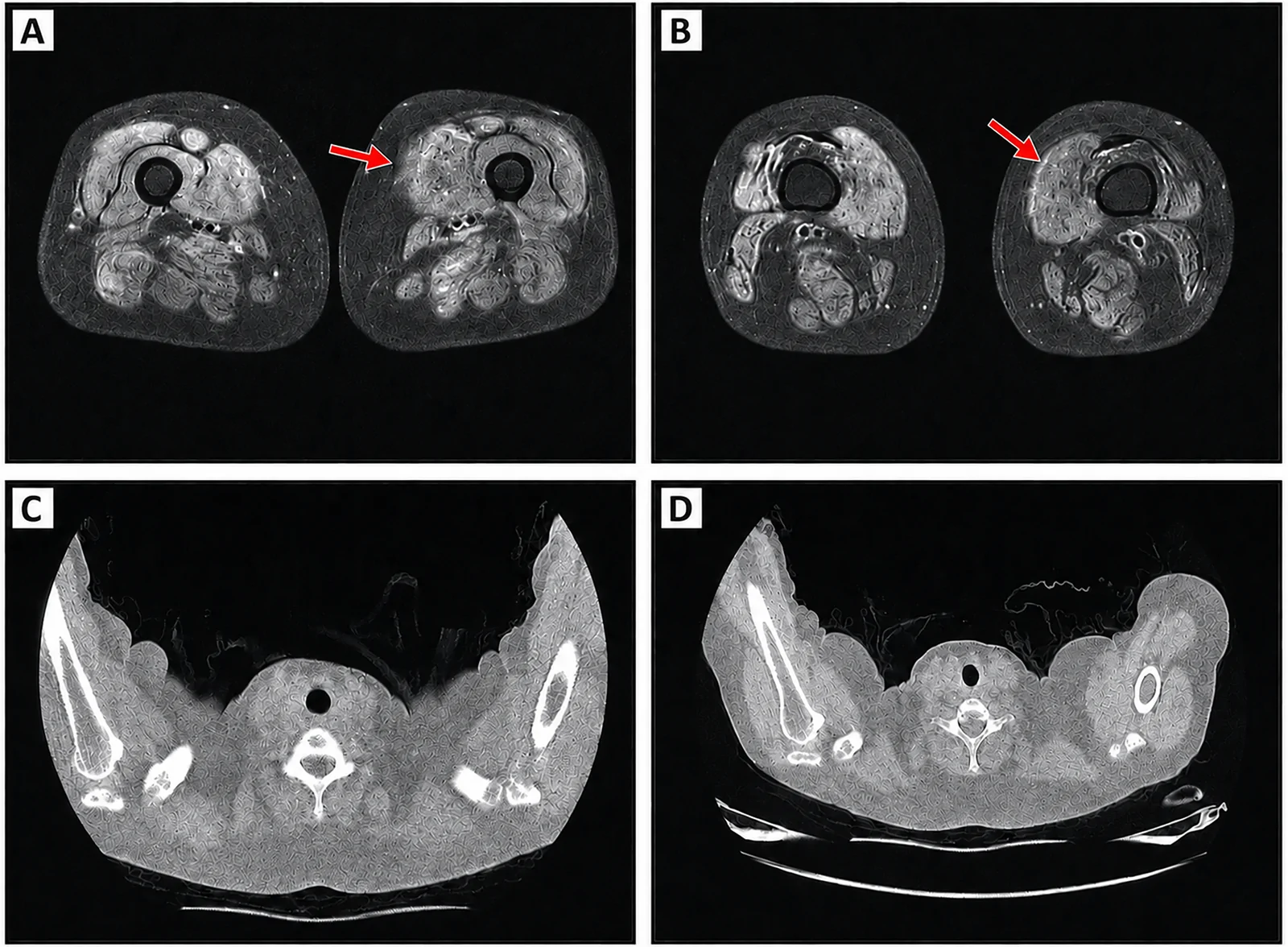
Representative imaging findings. **(A)** Axial thigh MRI obtained approximately 6 weeks before the index admission. **(B)** Axial thigh MRI obtained 2 days after admission. Red arrows indicate areas of abnormal signal within the bilateral thigh musculature. **(C)** Axial non-contrast chest CT obtained approximately 6 weeks before admission (original slice thickness, 5.0 mm). **(D)** Thin-section axial chest CT obtained 4 days after admission. The corresponding chest CT examinations showed no extensive pulmonary parenchymal abnormality sufficient to account for the severity of alveolar hypoventilation.

An outside muscle biopsy had been recorded as compatible with dermatomyositis. Because the biopsy was performed at a geographically distant hospital, the patient reported that she could not retrieve the original report or slides. The value of repeat biopsy was explained to the patient and her family, but they declined another invasive procedure. No typical dermatomyositis rash was documented.

The myositis autoantibody panel used in this patient included MDA5, PL-12, PL-7, Jo-1, EJ, OJ, KS, Mi-2, TIF1-γ, NXP2, SRP, SAE, PM-Scl-75, PM-Scl-100, Ku, Ro-52, and HMGCR. Anti-SRP and the other tested antibodies were negative, whereas anti-HMGCR was detected at low levels on two occasions (0.35 and 0.27 U/mL; positivity cutoff >0.15 U/mL) (12). Low-level anti-HMGCR positivity raised the possibility of anti-HMGCR-associated immune-mediated necrotizing myopathy (IMNM). However, the overall phenotype was atypical for classic anti-HMGCR myopathy, given the modest peak CK elevation, prominent diaphragmatic and cardiac involvement, and persistent strong AMA-M2 positivity. Moreover, the original muscle-biopsy report and slides were unavailable for independent assessment of a necrotizing myopathy pattern. We therefore retained the broader diagnosis of AMA-M2-positive inflammatory myopathy with low-level anti-HMGCR positivity rather than classifying the patient definitively as anti-HMGCR-associated IMNM.

Using the 2017 EULAR/ACR criteria without biopsy variables, the aggregate score was 7.0 (age at onset >=40 years, symmetric proximal lower-limb weakness, neck flexors weaker than extensors, proximal legs weaker than distal legs, and elevated muscle enzymes), corresponding to an estimated IIM probability of 62.5% and probable IIM (13). These criteria support classification as IIM but do not independently determine the IMNM subtype. In conjunction with the unavailable outside pathology, this supported retaining the broader clinical designation AMA-M2-positive inflammatory myopathy with low-level anti-HMGCR positivity.

AMA-M2 was repeatedly strongly positive (>200 RU/mL), with concurrent anti-gp210 and anti-sp100 positivity. The patient had persistent cholestatic biochemical abnormalities dominated by elevated gamma-glutamyl transferase and alkaline phosphatase, while abdominal imaging showed no biliary obstruction. She therefore fulfilled two established diagnostic domains for PBC-biochemical cholestasis and PBC-specific autoantibodies-and, according to EASL/AASLD guidance, liver biopsy was not required to establish the clinical diagnosis of PBC (14, 15). Early aminotransferase elevations were not used as diagnostic evidence for PBC. Subsequent testing for autoimmune hepatitis-associated antibodies, including anti-smooth muscle, anti-LKM-1, and anti-SLA/LP antibodies, was negative, and the available biochemical and immunologic findings did not support concomitant autoimmune hepatitis. PBC-AIH overlap was therefore considered clinically unlikely. The absence of liver biopsy mainly limited further histopathologic characterization and did not affect the clinical diagnosis of PBC.

### 2.3 Respiratory muscle and diaphragmatic involvement

Approximately 7 weeks before admission, respiratory function deteriorated rapidly. Arterial blood gas analysis showed pH 7.278 and PaCO2 94.6 mmHg; PaCO2 remained 73.9 mmHg approximately 3 days later, requiring continuous noninvasive ventilation (NIV). Repeated chest computed tomography showed no extensive pulmonary parenchymal abnormality or definite interstitial lung disease sufficient to explain the severity of alveolar hypoventilation (**Figures 1C, D**). Serial diaphragm ultrasonography, in contrast, demonstrated marked dysfunction. Early measurements showed a maximal inspiratory excursion of only 0.6-0.7 cm on the right. During the index admission, both hemidiaphragms were thin; the right diaphragm thickening fraction (TF) was 22.2% with a maximal inspiratory excursion of 1.0 cm, compared with 33% and 4.5 cm on the left. The combination of severe hypercapnia, restrictive ventilatory physiology, relatively minor parenchymal lung abnormalities, and direct evidence of diaphragmatic dysfunction favored respiratory muscle weakness as the principal mechanism of ventilatory failure.

### 2.4 Cardiac involvement

Cardiac abnormalities were concordant across electrical, biochemical, and imaging domains. The outside hs-cTnI concentration approximately 7 months before the index admission was 0.487 ng/mL (reference range, 0-0.0387 ng/mL). At our institution, hs-cTnI was 0.0256 ng/mL at the index admission and remained above the local upper reference limit of 0.0116 ng/mL at approximately 1 and 3 weeks (0.0169 and 0.0214 ng/mL, respectively). Although both measurements were hs-cTnI, the outside and in-house values were obtained using different assay systems and reference ranges; therefore, their absolute concentrations were not interpreted as a directly comparable quantitative trajectory. BNP was repeatedly elevated and reached 371 pg/mL during early follow-up. By approximately 4 months, hs-cTnI had normalized to 0.0048 ng/mL, whereas BNP remained 194 pg/mL. In IIM, cardiac troponin I is more specific for myocardial injury than cardiac troponin T, but troponin elevation itself does not establish an inflammatory etiology; electrocardiography, echocardiography, strain imaging, coronary assessment, and cardiac magnetic resonance imaging (CMR) provide complementary information (16, 17).

Atrioventricular conduction abnormalities had already been documented, and repeat ambulatory electrocardiography approximately 3 weeks after admission recorded 2,629 premature atrial contractions, 4,824 premature ventricular contractions, and four short runs of atrial tachycardia. LVEF remained preserved, but more sensitive imaging measures were abnormal. Echocardiography during the index admission showed LVEF 64% with persistent hypokinesis of the basal inferior wall. GLS was approximately -14.7% 1 month before admission and -15.4% during the index admission. Estimated pulmonary artery systolic pressure was approximately 29 mmHg. CMR and coronary evaluation were recommended but declined. The available findings therefore support cardiac involvement but do not establish myocarditis. In particular, the regional basal inferior wall-motion abnormality could reflect ischemic heart disease, prior injury, or another structural cardiac process, and an ischemic contribution cannot be excluded in the absence of coronary evaluation.

### 2.5 Treatment and longitudinal outcome

During the acute hypercapnic phase, the patient received methylprednisolone 40 mg/day, IVIG 20 g/day for 3 days, and NIV. Mycophenolate mofetil and subsequently tacrolimus were discontinued because liver tests worsened. PBC was treated with ursodeoxycholic acid, later supplemented with bezafibrate and liver-directed supportive therapy.

Because diaphragmatic and cardiac abnormalities persisted and steroid-sparing options were limited by liver dysfunction, rituximab 600 mg was given at approximately 1 and 3 weeks after admission. Hypercapnia had already begun to improve with glucocorticoids, IVIG, NIV, and supportive care before rituximab; recovery therefore cannot be attributed to rituximab alone.

CK and organ-specific indices followed different trajectories (**Table 2**). CK was 210 U/L at admission and 107 and 128 U/L at approximately 1 and 3 weeks, respectively, while hs-cTnI remained elevated at 0.0169 and 0.0214 ng/mL and BNP was 130 and 371 pg/mL.

**Table 2:** Representative longitudinal biochemical and cardiopulmonary findings.

Relative time	CK (U/L)	hs-cTnI (ng/mL)	BNP (pg/mL)	PaCO2 (mmHg)	Key organ-specific findings
T - ~7 months	785	0.487*	–	–	Outside hs-cTnI markedly elevated relative to its assay-specific reference range; arrhythmias and conduction abnormalities already present.
T - ~8 weeks	547	0.0238	505	–	Cardiac biomarkers were already elevated before the major ventilatory deterioration.
T - ~7 weeks	579	–	480.8	94.6	Severe hypercapnic respiratory failure; NIV initiated.
T - ~6 weeks	345	–	176.1	73.9	Marked hypercapnia persisted despite falling CK.
T - ~5 weeks	212	–	110.5	35.8	Gas exchange improved after immunotherapy and NIV.
T0	210	0.0256	161	–	Right diaphragm TF 22.2%, excursion 1.0 cm; LVEF 64% with regional wall-motion abnormality and reduced GLS.
T + ~1 week	107	0.0169	130	–	CK normal; hs-cTnI and BNP remained abnormal.
T + ~3 weeks	128	0.0214	371	–	Cardiac biomarker abnormalities persisted despite normal CK.
T + ~4 months	161	0.0048	194	46.9	CK and hs-cTnI normal; diaphragm TF 10% right and 11% left; FVC 51% and TLC 57.5% predicted.
T + ~7 months	–	–	187.52	–	Basal inferoposterior wall hypokinesis and grade II diastolic dysfunction remained. No repeat PFT/ABG available.

*Outside hs-cTnI at T - ~7 months: reference range 0-0.0387 ng/mL. The outside and in-house hs-cTnI measurements used different assay systems/reference ranges and were not interpreted as directly quantitatively comparable. The local upper reference limit for in-house hs-cTnI was 0.0116 ng/mL. CK normalization was defined according to the laboratory report at each time point. A dash indicates that the variable was not available. No repeat PFT or arterial blood gas was available at the approximately 7-month admission.

At approximately 4 months, CD19-positive B cells were 0.1% (6 cells/uL). AMA-M2 remained 196 RU/mL, anti-gp210 positive, and anti-sp100 weakly positive. CK 161 U/L and hs-cTnI 0.0048 ng/mL were normal, while BNP was 194 pg/mL. Room-air blood gas showed pH 7.431, PaCO2 46.9 mmHg, PaO2 70.3 mmHg, and bicarbonate 30.0 mmol/L. Home NIV had not been fully discontinued. Diaphragm TF was 10% right and 11% left, with maximal excursions 0.8 and 3.4 cm; FVC was 51.0% predicted and TLC 57.5% predicted.

At approximately 7 months, BNP was 187.52 pg/mL; echocardiography still showed basal inferoposterior hypokinesis and grade II diastolic dysfunction. CD19-positive B cells remained 0%. No repeat pulmonary function test or arterial blood gas was available. A further rituximab 600 mg infusion was given, with prednisone 15 mg/day continued.

## 3 Discussion

The main contribution of this case is the disproportion and temporal dissociation between conventional skeletal muscle measures and organ involvement. The patient remained independently ambulatory, had MMT8 76/80, and never had CK above 800 U/L, yet developed life-threatening hypercapnic respiratory failure with diaphragmatic dysfunction, conduction disease, atrial and ventricular arrhythmias, abnormal cardiac biomarkers, and impaired myocardial strain. Limb strength and CK alone would therefore have underestimated disease burden.

The phenotype is consistent with emerging descriptions of AMA-M2-associated myositis. Nishimori et al. (5) proposed a subgroup with chronic myopathy, IMNM-like pathology, and frequent cardiac and respiratory involvement, and Pang et al. (10) found AMA-M2 independently associated with cardiac involvement. Low-level anti-HMGCR positivity on two occasions raised the possibility of anti-HMGCR-associated IMNM. However, the modest peak CK elevation, prominent diaphragmatic and cardiac involvement, persistent strong AMA-M2 positivity, and lack of independently reviewable necrotizing pathology made the overall phenotype atypical for classic anti-HMGCR myopathy (12). We therefore regard anti-HMGCR as an additional serologic finding rather than sufficient evidence to reclassify the entire case as anti-HMGCR-associated IMNM; conversely, its presence also argues against attributing the entire phenotype solely to AMA-M2.

Respiratory recovery was dynamic. PaCO2 reached 94.6 mmHg despite no extensive parenchymal lung disease. By 4 months, PaCO2 had improved to 46.9 mmHg, but FVC and TLC remained about half of predicted and diaphragm TF had fallen from 22.2% to 10% on the right and from 33% to 11% on the left. The reduction in TF at 4 months could reflect persistent muscle disease or atrophy, deconditioning, glucocorticoid-related myopathy, and/or measurement variability. Serial diaphragm ultrasonography is effort- and technique-dependent. Although the same ultrasound equipment model was used across follow-up assessments, examinations were performed by different operators; differences in respiratory effort, probe position, and operator technique may therefore have contributed. No technically comparable later diaphragm measurement was used for quantitative longitudinal interpretation. The later FVC/TLC trajectory is unknown because PFT was not repeated.

Cardiac involvement was supported across electrical, biochemical, and imaging domains. Preserved LVEF did not exclude reduced GLS or regional wall-motion abnormality, and hs-cTnI elevation accompanied conduction disease and atrial/ventricular arrhythmias. cTnI is more myocardium-specific than cTnT in IIM, but myocardial injury is not synonymous with myocarditis (16, 17). Without CMR or coronary assessment, the basal inferior/inferoposterior abnormality cannot be specifically attributed to inflammation; ischemic or other structural causes remain possible. At approximately 7 months, BNP remained 187.52 pg/mL with persistent regional hypokinesis and grade II diastolic dysfunction. A 2025 review found deterioration of arrhythmias and/or cardiac function in 41.7% of immunosuppressed AMA-positive myositis cases and more frequent device therapy among those with worsening cardiac lesions, supporting early detection and serial cardiology follow-up (18).

The clinically important observation is therefore not a static persistent deficit but asynchronous recovery. CK normalized early; by 4 months hs-cTnI and gas exchange had also improved, while restrictive physiology and diaphragmatic dysfunction remained. At approximately 7 months, cardiac abnormalities persisted. Because PFT and blood gases were not repeated then, later respiratory physiology cannot be fully defined. CK reflects active skeletal myofiber injury but not chronic atrophy, diaphragmatic contractility, conduction-system disease, or structural myocardial injury; normal CK should not be equated with multisystem remission.

After rituximab, peripheral CD19-positive B cells were markedly depleted while AMA-M2 remained strongly positive and organ recovery was heterogeneous. This single case cannot establish AMA-M2 pathogenicity or treatment responsiveness, but it shows that B-cell depletion, antibody titer, and organ recovery were not synchronous. Recovery also cannot be attributed to rituximab alone because hypercapnia improved before the first dose; prior studies likewise suggest differing skeletal-muscle and cardiac responses to immunosuppression (11, 19, 20).

PBC provided both autoimmune context and a treatment constraint. Alkaline-phosphatase-predominant cholestasis, strongly positive AMA-M2 with anti-gp210/anti-sp100, and absence of biliary obstruction met clinical/serologic criteria for PBC (14, 15). Early aminotransferase elevations were assessed separately and were not used as diagnostic evidence for PBC. Autoimmune-hepatitis-associated antibodies, including anti-smooth muscle, anti-LKM-1, and anti-SLA/LP, were negative, and the available biochemical and immunologic findings did not support concomitant autoimmune hepatitis; PBC-AIH overlap was therefore considered clinically unlikely. The lack of liver biopsy limited histopathologic characterization but did not undermine the clinical diagnosis of PBC. PBC did not explain the myopathic electromyography, muscle MRI abnormalities, diaphragmatic dysfunction, or cardiac findings, while liver dysfunction limited sustained use of mycophenolate and tacrolimus.

Limitations include unavailable outside biopsy material and refusal of repeat biopsy; low-level anti-HMGCR serologic overlap; absence of CMR and coronary evaluation; and incomplete respiratory-muscle physiology. Serial diaphragm ultrasound is effort- and technique-dependent. Although the same ultrasound equipment model was used across follow-up assessments, examinations were performed by different operators, and strict technical comparability could not be ensured. Later diaphragm-ultrasound data were therefore not used for quantitative longitudinal interpretation. PFT and arterial blood gases were not repeated at the approximately 7-month admission. Nevertheless, the combined longitudinal data from blood gases, diaphragm ultrasonography, PFT, ambulatory electrocardiography, hs-cTnI/BNP, and cardiac imaging consistently show that CK normalization and recovery of critical organ function were not synchronous.

## 4 Conclusion

AMA-M2-positive inflammatory myopathy may be underestimated by conventional skeletal muscle measures: modest limb weakness and CK elevation can coexist with clinically important diaphragmatic and cardiac abnormalities. In this patient, recovery was dynamic and organ-specific: gas exchange improved early, while restrictive physiology and bilateral diaphragmatic dysfunction remained at approximately 4 months and cardiac abnormalities persisted at later follow-up. Low-level anti-HMGCR positivity indicated serologic overlap but did not establish anti-HMGCR-associated IMNM without confirmatory pathology. CK normalization should therefore not by itself reduce follow-up intensity; rhythm surveillance, cardiac biomarkers/imaging, blood gases, pulmonary function, and direct respiratory-muscle assessment should be guided by the clinical phenotype.

## Data Availability

The datasets presented in this study can be found in online repositories. The names of the repository/repositories and accession number(s) can be found in the article/supplementary material.
